# Combining parenting and economic strengthening programmes to reduce violence against children: a cluster randomised controlled trial with predominantly male caregivers in rural Tanzania

**DOI:** 10.1136/bmjgh-2020-002349

**Published:** 2020-07-08

**Authors:** Jamie Lachman, Joyce Wamoyi, Thees Spreckelsen, Daniel Wight, Jane Maganga, Frances Gardner

**Affiliations:** 1 Department of Social Policy and Intervention, University of Oxford, Oxford, UK; 2 MRC/CSO Social and Public Health Sciences Unit, University of Glasgow, Glasgow, UK; 3 National Institute for Medical Research Mwanza Research Centre, Mwanza, Mwanza, United Republic of Tanzania; 4 School of Social and Political Sciences, University of Glasgow, Glasgow, Glasgow, UK

**Keywords:** child health, prevention strategies, cCluster randomised trial

## Abstract

**Introduction:**

Parenting programmes may reduce the risk of violence against children and improve child well-being. However, additional economic support may be necessary in highly deprived rural communities in sub-Saharan Africa. Furthermore, delivering programmes within farmer groups may increase male caregiver recruitment and engagement.

**Methods:**

A parallel cluster randomised controlled trial examined the combined and separate effects of parenting and economic strengthening programmes on reducing violence against children aged 0–18 years in farming communities in Tanzania (n=248 families; 63% male caregivers). Eight villages were randomly assigned to four conditions (2:2:2:2): (1) 12-session parenting programme (n=60); (2) agribusiness training (n=56); (3) parenting and agribusiness combined (n=72); (4) control (n=60). Parent-report, child-report and early childhood observation assessments were conducted at baseline, mid-treatment and post-treatment. Primary outcomes were child maltreatment and parenting behaviour. Secondary outcomes included corporal punishment endorsement, parenting stress, parent/child depression, child behaviour, economic well-being and child development.

**Results:**

At post-treatment, parents and children receiving the combined interventions reported less maltreatment (parents: incidence rate ratio (IRR=0.40, 95% CI 0.24 to 0.65; children: IRR=0.40, 95% CI 0.17 to 0.92). Parents reported reduced endorsement of corporal punishment (*D_w_*=−0.43, 95% CI −0.79 to 0.07) and fewer child behaviour problems (*D_w_*=−0.41, 95% CI −0.77 to 0.05). Parents in parenting-only villages reported less abuse (IRR=0.36, 95% CI 0.21 to 0.63) and fewer child behaviour problems (*D_w_*=−0.47, 95% CI −0.84 to 0.11). Parents in agribusiness-only villages reported fewer child behaviour problems (*D_w_*=−0.43, 95% CI −0.77 to 0.08) and greater household wealth (*D_w_*=0.57, 95% CI 0.08 to 1.06). However, children in agribusiness-only villages reported increased physical abuse (IRR=2.26, 95% CI 1.00 to 5.12) and less positive parenting (*D_w_*=−0.50, 95% CI −0.91 to 0.10). There were no other adverse effects.

**Conclusion:**

Parent training may be the active ingredient in reducing maltreatment in farmer groups with majority male caregivers, while agribusiness training programmes may have unintended negative consequences on children when delivered alone. Locating parenting support in existing farmer groups can engage much higher proportions of fathers than stand-alone programmes.

ClinicalTrials.gov: NCT02633319

**Significance of this study**

**What is already known?**

- Over 1 billion children experience violence each year with a disproportionate number in low-income and middle-income countries (LMICs).
- Systematic reviews suggest that parenting interventions may reduce violence against children in LMICs, but that families living in highly deprived, underserved low-income communities may require male participation.
- Reviews also suggest that while economic strengthening interventions may reduce household poverty, there is limited evidence on their impact on reducing violence against children, with some evidence suggesting potential harm.

**What are the new findings?**

- The parenting intervention delivered to farmer groups was effective at reducing violence against children, with or without an economic strengthening component.
- A high percentage of male caregivers were recruited to the parenting programme when delivered through existing farmer groups.
- In villages that only received an agricultural intervention, children reported increased physical abuse and reduced positive parenting.

**What do the new findings imply?**

- Locating parenting interventions within existing farmer groups may increase recruitment and engagement of fathers and other male caregivers.
- Parenting interventions delivered to mixed-gender groups with a large proportion of male caregivers may be effective at reducing violence against children in highly deprived rural communities in LMICs.
- While agricultural economic strengthening programmes may increase economic security, they should be combined with parenting programmes in order to reduce the risk of additional harm to children.

## Introduction

### Background on maltreatment in LMICs

Child maltreatment and other childhood adversities occur in many low-income and middle-income countries (LMICs) at higher rates than in high-income countries (HICs)—rates that often exceed 50%.^1^ In Tanzania, a national survey found that over 70% of respondents aged between 13 and 24 years had experienced physical violence before the age of 18.^2^ The survey also identified a correlation between physical, emotional and sexual violence against children; approximately 80% of respondents who experienced sexual violence also experienced physical violence as a child, and nearly all children who experienced physical violence also experienced emotional violence. Parents and other adult relatives were the most commonly reported perpetrators, with corporal punishment considered a norm perpetuated by other cultural values related to gender, family privacy, male honour and expectations of child respect and obedience.^2^

Recent studies have highlighted the long-term and far-reaching consequences of violence against children, including serious physical and mental health problems later in life, as well as difficulties in school, employment and relationships.^3^ Child maltreatment can have substantial intergenerational effects, maltreated children being more likely to maltreat their own children.^4^ Child maltreatment is also a risk factor for later intimate partner violence, criminal activity, HIV infection, transactional sex and other negative health and mental health outcomes.^3 5^ Furthermore, child maltreatment has an economic impact relating to the treatment of victims’ health problems, criminal justice and welfare costs, and lower economic productivity.^6^

### Parenting programmes to prevent child maltreatment

Parenting programmes have shown particular promise in preventing child maltreatment and other childhood adversities.^7 8^ Parenting programmes typically aim to strengthen caregiver–child relationships through positive parenting and to help parents to manage child behaviour problems through effective, age-appropriate, non-violent discipline strategies. A meta-analysis reported that parenting programmes successfully reduced substantiated and self-reported child maltreatment and associated risks, both in high and middle-income countries.^8^ A systematic review demonstrated that parenting practices in sub-Saharan Africa are associated with the same pattern of child outcomes as in HICs.^9^ There is also promising evidence that parenting programmes can effectively reduce child maltreatment in LMICs.^10^

Despite emerging evidence of the effectiveness of parenting interventions, local governments and service providers in LMICs face multiple challenges implementing such programmes.^11^ Transported parenting programmes may not fit local contexts and may require cultural adaptation to be relevant to local families. Parents, especially fathers/male caregivers, may require specific targeting to overcome barriers to participation, including identifying entry points for programme delivery that harness existing social groups.^12^ Additional components may also be necessary to address the consequences of poverty, particularly relevant in highly deprived rural communities in sub-Saharan Africa that largely rely on subsistence agriculture with little access to economic development.

### Economic strengthening programmes

Poverty has been identified as a major risk factor for physical and emotional child abuse.^13^ Consequently, economic strengthening programmes have been highlighted as an important component in the reduction of violence against children, particularly in multiply deprived and impoverished families.^14^ While there is some evidence that these programmes may reduce risks of abuse and exploitation, the evidence of their effectiveness when delivered alone is limited.^15^ Furthermore, economic strengthening programmes may have unintended harmful effects on children; a systematic review found that at least one negative child outcome in 20% of the 46 identified trials.^16^ At the same time, there is emerging empirical evidence suggesting the benefits of an integrated approach that combines both parenting and economic strengthening programmes.^16^ For instance, a recent trial in Burkina Faso found reduced harsh parenting and abuse in villages where women received an economic support package combined with a family-based intervention in comparison to those which only received the economic intervention.^17^ However, this study did not examine whether delivering the family-based intervention alone was sufficient to reduce child maltreatment and improve parent–child relationships. A recent individual participant data meta-analysis of parenting programme effectiveness in HICs found no differential effects by family socioeconomic status.^18^

The Skilful Parenting and Agribusiness Child Abuse Prevention Study aimed to investigate the inter-relationship between economic strengthening programmes and parent management training in reducing child maltreatment. This is of particular importance given the need for combined interventions that address multiple Sustainable Development Goals (SDGs) across economic, social and environmental domains.^19^ Combining parenting and economic strengthening may have a positive impact beyond SDG targets 16.2 (ie, eliminating violence against children) and 5.2 (ie, eliminating violence against women and girls). Additional effects on SDG targets may include ending poverty (1.1, 1.2, 1.4. 1.5) and hunger (2.1, 2.2, 2.3, 2.4, 2.A), and sustaining income growth (10.1) through increased agricultural production and food security. Therefore, we hypothesised that both approaches would reduce child maltreatment, but a combined intervention would have more robust effects across multiple outcomes associated with increased risk of violence against children.

## Methods

### Study design

We used a cluster randomised controlled trial (RCT) to examine the combined and separate effects of parenting and economic strengthening programmes on reducing child maltreatment in rural Tanzania (Pan African Clinical Trials: PACTR201610001267268, preregistered 14 September 2015; ClinicalTrials.gov: NCT02633319, registered at end baseline data collection on 14 December 2015). Reporting follows the CONSORT extension for cluster RCTs (online supplementary tables 6 and 7).

10.1136/bmjgh-2020-002349.supp1 Supplementary data

### Setting

The study was conducted in the Shinyanga Rural District, populated primarily by Sukuma-speaking communities mainly dependent on subsistence-level agriculture. Eight villages geographically isolated from each other by at least 20 km were selected in collaboration with the Tanzanian Ministry of Agriculture, Food Security, and Cooperatives. Existing village-based, government-organised farmer groups served as delivery platforms for both the parenting and agribusiness interventions. Two farmer groups were selected within each participating village using a random selection procedure at public community meetings in which the research investigators selected different-coloured beads from a concealed box in the presence of farmer group leaders (n=16 farmer groups).

### Participants

Eligible parents and caregivers (n=248) were recruited from families who were members of the selected farmer groups in each village (8–22 parents per farmer group). Inclusion criteria were: (1) age 18 or older, (2) primary caregiver of a child in the household aged 3–17, (3) lived in household ≥4 nights per week, (4) registered member of agricultural farmer group and (5) provided consent to participate. We recruited child respondents aged 10–17 years from all participating families (n=176). If there was more than one child aged 10–17 in the household, the participating adult was asked to identify the child with whom they were having the most difficult relationship. Inclusion criteria for child respondents included: (1) aged 10–17 years, (2) lived in the house ≥4 nights per week, (3) caregiver participating in the study and (4) adult and child provided consent. Finally, we conducted observational assessments on early child development in participating families where there was a child aged 1–36 months, using a random number generator to avoid selection bias by parents (n=134). Adults and children were excluded if they exhibited acute mental health problems (none met this criterion). Families received a bar of soap after each assessment as compensation. Throughout this paper ‘parent’ is intended to include adult caregivers of children who are not biological parents.

### Interventions: skilful parenting and agribusiness

**Figure 1:**
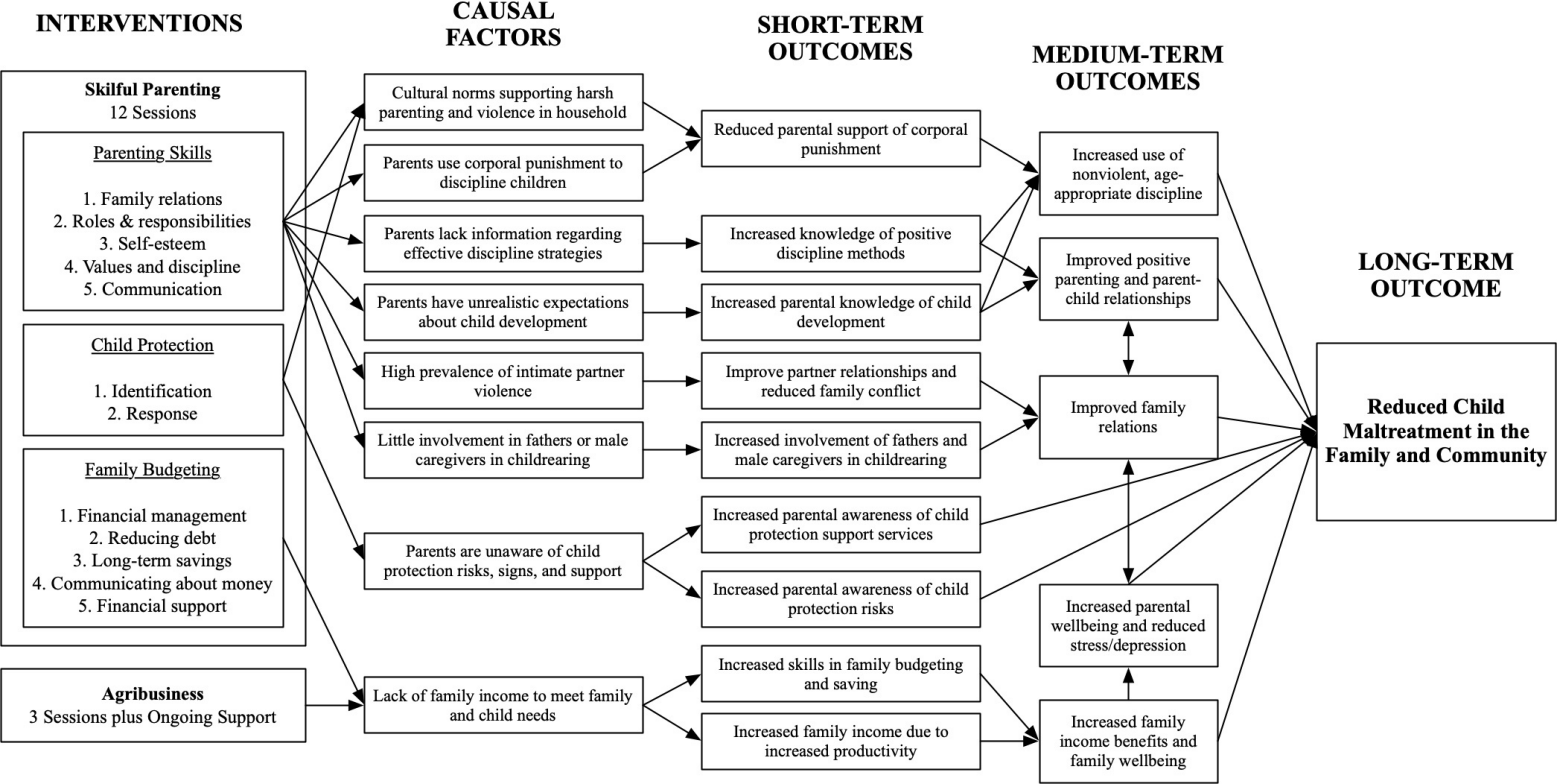
Theory of change model for combined skilful parenting plus agribusiness training interventions approximately here.

We examined the differential and combined effects of two community-based interventions—a parenting and family budgeting programme and an agribusiness training programme—with the overall goal to reduce child maltreatment by improving parenting behaviours and reducing family stress due to food insecurity and financial hardship (figure 1). Skilful parenting is a 12-session group-based programme consisting of five sessions on parenting skills, two on child protection and five on family budgeting. Originally developed in Kenya and adapted for Tanzania, it was delivered to farmer groups by Kiswahili-speaking professional trainers from Investing in Children and Societies, an international non-profit organisation (www.icsafrica-sp.org) with local offices in Shinyanga. The agribusiness programme targets food and income insecurity by providing smallholder farmer groups access to drought-resistant seeds, credit for farm inputs, advice to improve farming techniques and market connections. The intervention is delivered to farmer group members by trained staff from a local economic enterprise initiative working in collaboration with the Ministry of Agriculture over three intensive workshops during the planting season and ongoing support through harvesting season.

### Randomisation and masking

Cluster randomisation was conducted at village level prior to baseline assessments, to reduce the likelihood of contamination between arms. An external researcher used concealed computer-generated codes to randomly allocate eight villages into three treatment arms and a control arm (two villages per arm): (1) agribusiness plus parenting (n=72), (2) agribusiness-only (n=56), (3) parenting-only (n=60) and (4) control (n=60). The implementing partner notified the participating families of their allocation status after baseline data collection. The implementing partner also committed to providing the agribusiness, parenting or both interventions to all villages after the end of the study. The allocation status of other participating villages was concealed from selected villages, thus reducing the potential for intervillage rivalries. Research assistants conducting assessments were only blind to allocation at baseline; however, the data analyst was blind to allocation until after initial intention-to-treat analyses.

### Power calculations

Due to funding constraints and the need to use village-level cluster randomisation, the sample size was limited. Post-hoc calculations suggested 7% power to detect a large, clustering-adjusted *D_w_*=0.7, effect (with alpha=0.05, m=2 cluster per condition, n=60 participants per condition, intracluster correlation=0.1).^20^

### Procedure

Data collection occurred at three stages: (1) baseline (September 2015), (2) mid-treatment after the parenting and child protection modules of the parenting intervention and before the harvesting season in the agribusiness intervention (June 2016) and (3) at post-treatment, 1 year from baseline (September 2016). Because the pattern of family life varies considerably with the cycle of planting and harvesting, it was important that both baseline and post-treatment assessments were conducted at the same time after harvesting. Data included parent-reported assessments for all families, child-reported assessments for families with children aged 10–17 and early childhood assessments for families with children aged 0–3. Questionnaires were translated into Kiswahili, and back-translated.

Trained research assistants used e-tablets to administer consent forms and questionnaires. In addition, we used audio computer-assisted self-interviewing technology to decrease stigma and increase responsivity for sensitive items regarding child maltreatment and intimate partner violence. Research assistants were also able to explain questions in Sukuma for families with poor understanding of Kiswahili. Post-treatment qualitative interviews and focus groups were also conducted to assess programme delivery and engagement; these will be reported in separate publications. The mid-treatment data collected (June 2016) is not used or reported on in this paper.

### Outcomes

#### Primary outcomes

Primary outcomes were parent-report and child-report of child maltreatment and parenting behaviour. Frequency of overall child maltreatment, as well as physical, emotional and sexual abuse, was measured using the ISPCAN Child Abuse Screening Tool-Trial (adult-report: 22 items; child-report: 24 items).^21^ The Alabama Parenting Questionnaire (adult-report: 37 items; child-report: 42 items) assessed parent involvement, positive interaction, poor supervision, inconsistent discipline and effective discipline (parent-report and child-report), as well as harsh discipline (child-report only). Items were summed for each subscale as well as a total positive parenting score by reversing negative items.

#### Secondary outcomes

Secondary outcomes included parent-report of parenting stress (Parenting Stress Scale, 18 items), parent depression (Centre for Epidemiologic Studies Depression Scale, 20 items) and exposure to intimate partner violence (Revised Conflict Tactics Scale Short Form, 10 items). We also measured parent-report and child-report of attitudes supporting corporal punishment (UNICEF Multiple Indicator Cluster Survey, 1 item), parent and child use of alcohol (1 item each) and child behaviour problems and prosocial behaviour (Strengths and Difficulties Questionnaire, 25 items). Five items assessing *heshima*, or respectful behaviour, were added to the prosocial scale as a culturally sensitive assessment of positive child behaviour. Child-reported depressive symptoms were measured using the 10-item Child Depression Inventory short form (10 items), and child-reported sexual behaviour in the past month was based on items from the South African National Survey of HIV and Risk Behaviour (15 items). Economic outcomes included household hunger (Household Hunger Scale; parent-report and child-report), child food consumption in the previous week (child-report), household assets (parent-report) and basic child necessities (child-report). Finally, early childhood outcomes for children aged 0–36 months included observational assessments of stimulation and responsiveness (HOME Inventory), child development (Ages and Stages Questionnaire) and infant/toddler growth based on measurements of weight and height.

In addition to the outcomes prespecified in our trial protocol, we also examined infant/child nutritional health based on measurements of mid-upper arm circumference, parents’ attitudes towards family budgeting, and agricultural production based on the number of 90 kg bags of maize from the previous harvest (parent-report only). These were analysed as post-hoc secondary outcomes.

All self-report outcomes using frequency ratings referred to the past month unless otherwise specified (online supplementary tables 1 and 2).

### Statistical analysis

This paper reports on primary and secondary outcomes at post-treatment using an intention-to-treat analyses. Linear random effects regression models were used to estimate treatment effects accounting for the nesting of participants in farmer groups and baseline differences between groups on primary and secondary outcomes. We did not adjust for differences between groups on demographic characteristics due to insufficient power. Standardised effect sizes (*D_w_*),^20^ and 95% CIs were obtained by dividing raw coefficients for each contrast by farmer group cluster. Incidence rate ratios (IRRs) and 95% CIs were estimated with a Poisson regression for count outcomes. To handle missing data we use multiple imputation with chained equations (R-package: *Mice*, V.3.6). Analyses were undertaken in R V.3.3.0. Borderline effect sizes of *D_w_*>0.40 or 95% CIs within ±0.10 of 0 were considered clinically relevant. The analysis syntax is available at https://osf.io/54r9p/

### Participant and public involvement statement

Representatives from the implementing organisation, Investing in Children and Our Societies, were closely involved in developing and refining the research questions, study design and ethical procedures. We met on a quarterly basis during the study to discuss ongoing implementation and emerging issues. At the end of the study, the implementing organisation and other stakeholders including the Government of Tanzania commented on the findings and contributed to the dissemination of results. Although it was difficult to involve study participants, we engaged with village and farmer group leaders at key points during the study to inform them of the process and outcomes.

## Results

**Figure 2:**
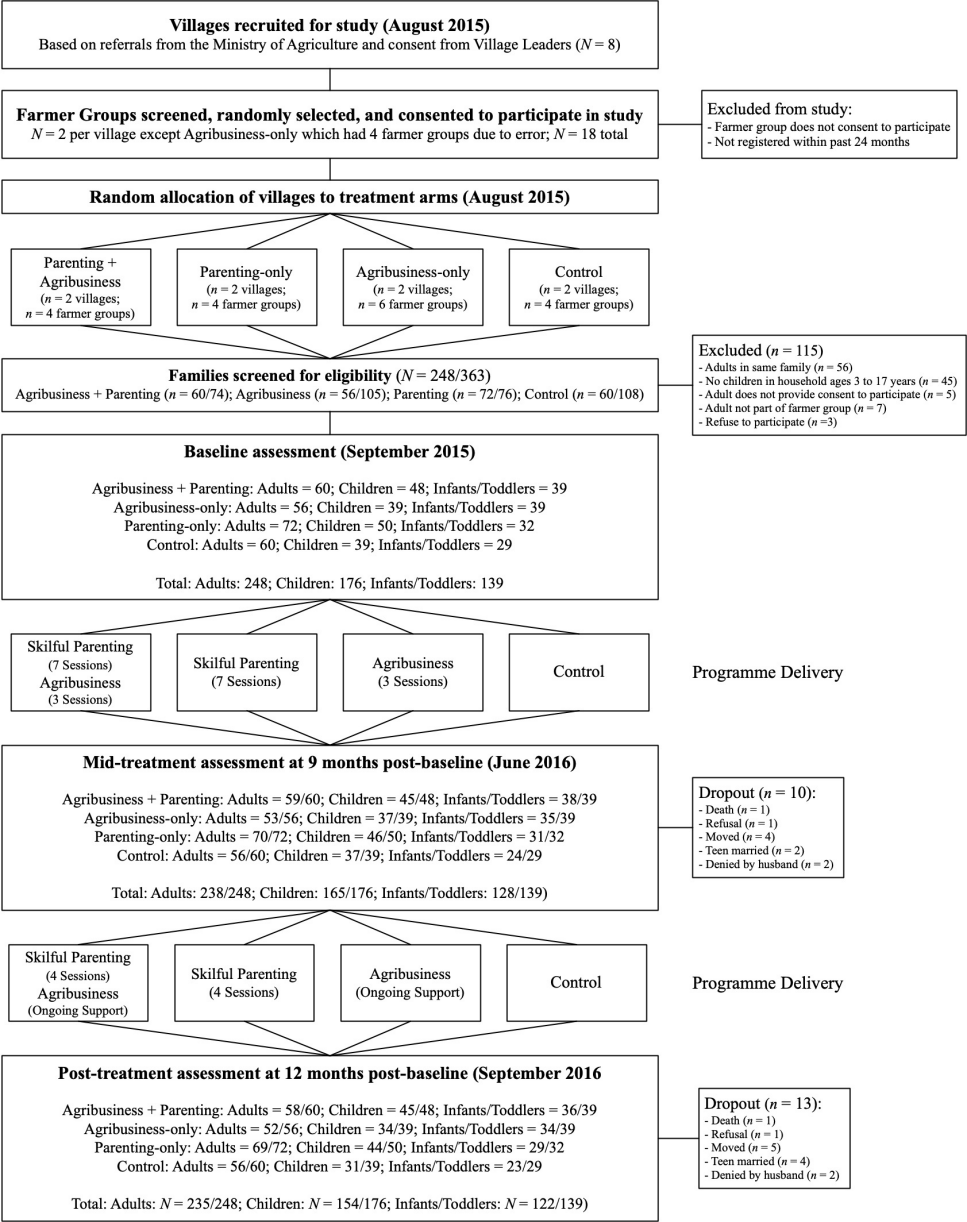
Trial profile.

Study retention was considerably higher than anticipated with 94.8% adults (n=235/248), 87.5% children (n=154/176) and 87.8% in their early childhood (n=122/139) assessments completed at post-treatment, with no differences between arms (figure 2). Due to delays in the implementation of the parenting intervention as a result of inaccessibility of villages during the rainy season, the final session on family budgeting was not delivered. Enrolment in parenting groups was 91% of those who completed baseline assessments, with an average attendance rate of 60% (7.5 out of 11 delivered sessions), with no significant differences in attendance between arms. The enrolment rate for agribusiness training was 85%, with an average attendance rate of two out of three total sessions. Parents in the combined intervention villages attended the agribusiness training sessions significantly less than those in the agribusiness-only villages (combined: M=1.80 sessions, SD 0.73; agribusiness-only: M=2.27, SD 0.69, *t*=−3.46, p=0.001).

Approximately two-thirds of adult participants were fathers or other male caregivers (63% total male caregivers: 50% fathers, 6% grandfathers and 7% other relationship), primarily due to men being identified as the family representative to farmer groups. Children were 42% female, and 73% lived with at least one biological parent, 16% with either a grandparent or great-grandparent and 11% with an alternative caregiver (eg, step-parent, aunt, brother). Despite data collection being just after harvest, generally the most affluent time of year, 53% of adults reported having borrowed money to meet basic needs in the past month, and 29% reported consuming two or less meals per day. Twenty-six per cent of adults reported not having completed primary education, and 18% were illiterate. Sixty per cent of the children were currently enrolled in school and 52% could not read or had difficulty reading. More than 70% of the parents reported having experienced some form of physical abuse as a child (table 1).

**Table 1:** Characteristics of the sample at baseline

	Total	A+P	A only	P only	Control
Age of participants, M (SD)					
Adult*	43.12 (12.71)	41.65 (10.47)	44.93 (13.25)	40.64 (12.76)	44.61 (13.75)
Teen†	13.41 (2.01)	13.76 (1.87)	13.04 (2.18)	13.29 (1.81)	13.62 (2.09)
Child‡	11.18 (3.91)	12.05 (3.82)	10.60 (4.00)	11.23 (3.59)	10.95 (4.11)
Infant (months)§	17.94 (9.84)	19.11 (8.08)	18.44 (9.62)	18.46 (10.34)	15.28 (11.40)
Sex of participants, male, n (%)					
Adult***	157 (63.3)	33 (55.0)	63 (87.5)	27 (48.2)	34 (56.7)
Teen	104 (60.5)	31 (68.9)	32 (64.0)	18 (47.4)	23 (59.0)
Child	145 (58.5)	37 (61.7)	40 (55.6)	32 (57.1)	36 (60.0)
Infant**	71 (51.4)	23 (60.3)	13 (40.6)	14 (35.9)	21 (72.4)
Parent–child relationships: biological child, n (%)					
Teen	125 (72.7)	37 (82.2)	36 (72.0)	27 (71.1)	25 (64.1)
Child	180 (72.6)	49 (81.7)	50 (69.4)	40 (71.4)	41 (68.3)
Infant	98 (71.0)	30 (78.9)	22 (68.8)	28 (71.8)	18 (62.1)
Number of people per household, M (SD)	7.30 (3.42)	7.88 (3.16)	7.49 (4.61)	6.96 (2.86)	7.30 (3.43)
Adult marital status: married, n (%)	212 (85.5)	48 (80.0)	66 (91.7)	50 (89.3)	48 (80.0)
Adult not completed primary school, n (%)	65 (26.2)	24 (40.0)	14 (19.4)	13 (23.2)	14 (23.3)
Adult cannot read, or reads with difficulty, n (%)	61 (24.6)	23 (38.4)	10 (13.9)	11 (25.0)	14 (23.3)
Teen enrolled in school, n (%)	105 (61.0)	22 (48.9)	29 (58.0)	27 (71.1)	27 (69.2)
Child enrolled in school, n (%)	146 (58.9)	31 (51.7)	39 (54.2)	37 (66.1)	39 (65.0)
Parent experienced physical abuse as a child, n (%)	176 (71.3)	44 (73.3)	61 (84.7)	46 (82.1)	49 (83.1)

*Based on number of adult participants (n=248).

†Based on number of child respondents between ages 10 and 18 (n=176).

‡Based on total number of children reported by adults (n=248).

§Based on number of children aged 0–36 months (n=134); **p<.01, ***p<.001 indicating significant differences between arms based on χ^2^ tests.

Linear models and χ^2^ analyses examining equivalence among allocation arms at baseline found fewer female caregivers (χ^2^(df)=26.55(3), p<0.001) and fewer female children aged 0–36 months (χ^2^(df)=11.63(3), p<0.01) in the agribusiness-only villages and a higher proportion of adults who had high scores on the discipline outcome (χ^2^(df)=24.61(12), p=0.0016) in the control villages compared with the other arms.

At baseline, compared with the combined intervention, parents in the parenting intervention villages reported lower emotional (b=−2.42, p=0.003) and physical abuse (b=−1.99, p<0.001), and overall maltreatment (b=−3.81, p=0.010). Similarly parents in agribusiness-only villages reported lower agricultural assets (b=−7.746, p=0.033). The control arm had lower levels of physical abuse (b=−1.47, p=0.034) compared with the combined intervention arm. Children in agribusiness-only villages reported higher basic necessity scale scores (b=1.29, p=0.017) and higher positive parenting outcomes (b=7.27, p=0.028). Similarly, children in control villages reported higher basic necessity scores (b=1.05, p=0.049) and positive parenting outcomes (b=7.15, p=0.030). All other demographic characteristics and outcomes were equivalent at baseline.

### Intention-to-treat results

Intention-to-treat results comparing each of the intervention arms with controls at post-treatment including means and SD by allocation arm for primary outcomes are summarised in tables 2 and 3, and for secondary outcomes in online supplementary tables 3–5.

**Table 2:** Adult-report and child-report of child maltreatment outcomes based on the ISPCAN Child Abuse Screening Tool-Intervention (ICAST-I) using an intention-to-treat analysis and adjusting for differences at baseline

	Parent-report (n=248)					Child-report (n=176)				
	Pre M (SD)	Post M (SD)	IRR*	95% CI	ICC	Pre M (SD)	Post M (SD)	IRR*	95% CI	ICC
Overall child maltreatment					0.135					0.365
A+P	**7.19** (**9.93**)	**2.19** (**3.91**)	**0.40**	**0.24 to 0.65**		**12.93** (**20.92**)	**5.20** (**8.99**)	**0.40**	**0.17 to 0.92**	
A-only	**3.38** (**5.30**)	**2.71** (**5.55**)	**0.60**	**0.37 to 0.95**		11.38 (20.75)	15.45 (29.83)	1.29	0.62 to 2.70	
P-only	**6.43** (**10.56**)	**1.87** (**3.89**)	**0.36**	**0.21 to 0.63**		12.82 (24.01)	10.03 (19.61)	0.78	0.36 to 1.68	
Controls	4.63 (5.99)	4.79 (9.55)				5.62 (10.62)	8.52 (13.14)			
Physical abuse					0.126					0.241
A+P	**3.22** (**4.94**)	**0.74** (**1.74**)	**0.32**	**0.17 to 0.62**		3.80 (7.56)	1.31 (3.29)	0.50	0.19 to 1.30	
A-only	**1.24** (**2.18**)	**1.00** (**2.24**)	**0.52**	**0.28 to 1.00**		**2.94** (**6.94**)	**5.39** (**12.34**)	**2.26**	**1.00 to 5.12**	
P-only	**2.88** (**4.48**)	**0.77** (**1.62**)	**0.34**	**0.18 to 0.66**		4.55 (9.64)	2.21 (4.14)	0.99	0.34 to 2.35	
Controls	1.75 (2.93)	2.04 (4.08)				1.90 (5.50)	2.36 (5.00)			
Emotional abuse					0.083					0.386
A+P	**3.53** (**5.46**)	**0.71** (**1.69**)	**0.32**	**0.18 to 0.57**		**4.20** (**8.30**)	**1.51** (**3.46**)	**0.27**	**0.08 to 0.89**	
A-only	1.11 (1.78)	0.96 (1.84)	0.57	0.34 to 1.19		4.44 (7.81)	4.98 (11.50)	1.05	0.32 to 3.40	
P-only	**2.75** (**6.17**)	**0.52** (**1.52**)	**0.27**	**0.13 to 0.59**		4.50 (10.20)	4.88 (10.64)	0.92	0.30 to 2.88	
Controls	2.32 (4.02)	2.00 (5.15)				2.51 (5.46)	2.42 (4.49)			
Sexual abuse					0.169					†
A+P	0.08 (0.33)	0.02 (0.13)	3.69	0.33 to 40.85		0.03 (0.16)	0.26 (0.58)	0.66	0.25 to 1.76	
A-only	0.10 (0.42)	0.06 (0.29)	2.93	0.28 to 32.03		0.20 (0.53)	0.20 (0.55)	0.67	0.24 to 1.88	
P-only	0.07 (0.32)	0.13 (0.44)	5.85	0.51 to 66.48		0.24 (0.59)	0.15 (0.50)	0.46	0.14 to 1.52	
Controls	0.19 (0.55)	0.10 (0.41)				0.20 (0.50)	0.24 (0.61)			
Neglect					0.210					0.248
A+P	0.48 (0.95)	0.74 (1.83)	1.21	0.51 to 2.84		*4.93* (*7.43*)	*2.38* (*4.54*)	*0.44*	*0.18 to 1.08*	
A-only	1.03 (2.50)	0.75 (2.32)	0.72	0.31 to 1.71		4.00 (8.06)	5.09 (9.21)	1.31	0.58 to 2.92	
P-only	0.80 (1.96)	0.58 (1.51)	0.68	0.28 to 1.65		3.76 (7.21)	2.94 (5.61)	0.56	0.23 to 1.36	
Controls	0.57 (1.43)	0.75 (2.06)				1.21 (2.38)	3.74 (7.78)			

*IRR for child maltreatment outcome based on comparisons of postassessment scores for each treatment arm with the control arm using Poisson regression analyses and controlling for baseline differences. Significant effects are in bold (95% CI not crossing 1.00); borderline effects are in italics (95% CI crossover within ±0.10 of 1.00).

†No reliable ICC estimate possible.

A-only, agribusiness only; A+P, agribusiness plus parenting; ICC, intraclass correlation coefficient; IRR, incidence risk ratio; P-only, parenting only.

**Table 3:** Adult-report and child-report of parenting outcomes using an intention-to-treat analysis and adjusting for differences at baseline*

	Parent-report (n=248)					Child-report (n=176)			
	Pre M (SD)	Post M (SD)	ffect Size†	95% CI		Pre M (SD)	Post M (SD)	Effect Size†	95% CI
Total positive parenting					Total positive parenting				
A+P	*78.07* (*16.43*)	*84.81* (*14.93*)	*0.26*	*−0.10 to 0.62*	A+P	86.16 (15.45)	96.20 (14.21)	−0.05	−0.49 to 0.38
A-only	78.10 (16.10)	79.99 (15.63)	−0.03	−0.37 to 0.32	A-only	**87.58** (**15.35**)	**88.39** (**17.82**)	−**0.50**	−**0.91 to −0.10**
P-only	75.68 (14.81)	82.44 (18.90)	0.20	−0.17 to 0.56	P-only	93.42 (14.70)	98.44 (11.69)	−0.08	−0.50 to 0.34
Controls	75.87 (13.33)	79.34 (17.65)			Controls	93.31 (13.83)	99.10 (15.30)		
Positive involvement					Positive involvement				
A+P	*15.17* (*7.56*)	*18.74* (*8.58*)	*0.33*	*−0.03 to 0.69*	A+P	13.99 (7.73)	17.00 (8.71)	−0.18	−0.70 to 0.34
A-only	15.11 (8.50)	15.91 (9.32)	0.03	−0.32 to 0.37	A-only	*15.90* (*7.39*)	*15.15* (*10.42*)	*−0.47*	*−0.95 to 0.01*
P-only	*14.20* (*9.21*)	*18.01* (*11.0*)	*0.29*	*−0.08 to 0.65*	P-only	16.66 (8.36)	19.78 (7.38)	0.01	−0.49 to 0.51
Controls	15.20 (7.91)	15.69 (9.79)			Controls	17.26 (7.94)	19.66 (9.82)		
Positive interaction					Positive interaction				
A+P	*11.98* (*5.22*)	*13.84* (*5.38*)	*0.31*	*−0.05 to 0.67*	A+P	10.85 (5.31)	13.43 (6.52)	−0.00	−0.52 to 0.51
A-only	12.32 (5.01)	12.32 (5.32)	0.04	−0.30 to 0.38	A-only	11.92 (5.07)	11.83 (7.23)	−0.32	−0.79 to 0.15
P-only	11.71 (5.71)	12.22 (6.46)	0.04	−0.33 to 0.40	P-only	12.48 (5.04)	14.60 (4.89)	0.10	−0.39 to 0.59
Controls	11.13 (5.34)	11.91 (6.47)			Controls	12.90 (4.66)	14.01 (6.17)		
Poor supervision					Poor supervision				
A+P	9.47 (5.89)	8.37 (4.64)	−0.22	−0.57 to 0.14	A+P	1.37 (1.17)	1.00 (1.08)	−0.15	−0.63 to 0.34
A-only	9.79 (5.52)	9.01 (5.06)	−0.10	−0.44 to 0.24	A-only	1.08 (1.18)	1.01 (1.34)	−0.11	−0.56 to 0.35
P-only	10.64 (5.64)	8.59 (4.85)	−0.23	−0.59 to 0.14	P-only	0.79 (1.06)	0.91 (1.31)	−0.18	−0.65 to 0.29
Controls	10.53 (5.59)	9.64 (4.83)			Controls	1.05 (1.10)	1.15 (1.50)		
Inconsistent discipline					Inconsistent discipline				
A+P	7.55 (3.04)	7.22 (3.80)	0.30	−0.24 to 0.83	A+P	7.58 (2.89)	7.90 (2.93)	−0.13	−0.71 to 0.44
A-only	7.49 (2.98)	6.75 (3.95)	0.21	−0.31 to 0.72	A-only	9.02 (3.06)	7.84 (4.13)	−0.08	−0.61 to 0.44
P-only	7.82 (4.18)	6.69 (3.43)	0.18	−0.35 to 0.71	P-only	7.92 (3.11)	8.31 (3.38)	−0.02	−0.56 to 0.53
Controls	7.65 (3.65)	6.01 (3.59)			Controls	7.95 (2.87)	8.14 (4.04)		
Effective discipline					Effective discipline				
A+P	3.93 (2.38)	3.76 (2.04)	0.11	−0.25 to 0.47	A+P	3.92 (2.81)	3.13 (2.46)	−0.38	−0.88 to 0.12
A-only	3.94 (2.26)	3.70 (2.50)	0.08	−0.26 to 0.43	A-only	4.14 (3.34)	3.78 (3.95)	−0.17	−0.62 to 0.29
P-only	4.23 (3.40)	3.66 (2.29)	0.07	−0.29 to 0.44	P-only	3.80 (2.74)	3.90 (2.65)	−0.14	−0.62 to 0.33
Controls	3.72 (2.28)	3.50 (2.64)			Controls	3.54 (2.74)	4.28 (3.76)		
				Harsh parenting‡					
					A+P	5.23 (3.65)	2.81 (2.71)	−0.19	−0.82 to 0.45
					A-only	5.04 (3.91)	4.36 (3.74)	0.35	−0.23 to 0.93
					P-only	4.41 (3.25)	3.65 (2.51)	0.10	−0.50 to 0.69
					Controls	3.87 (3.19)	3.11 (2.74)		

*Alabama Parenting Questionnaire.

†Hedges *D_w_* effect sizes for parenting behaviour based on comparisons of postassessment scores for each treatment arm with the control arm using linear-mixed effects models with REML and controlling for baseline differences.

‡Harsh parenting subscale was child-report only;; Significant effects are in bold (95% CI not crossing 0); borderline effects are in italics (95% CI crossover within ±0.10 of 0).

A-only, agribusiness only; A+P, agribusiness plus parenting; P-only, parenting only; REML, restricted maximum likelihood.

### Primary outcomes

Parents reported significant reductions in overall child maltreatment in all three intervention groups (combined: IRR=0.40, 95% CI 0.24 to 0.65; parenting-only: IRR=0.36, 95% CI 0.21 to 0.63; agribusiness-only: IRR=0.60, 95% CI 0.37 to 0.95). Children in the combined villages also reported reductions in overall child maltreatment (IRR=0.40, 95% CI 0.17 to 0.92), whereas there were no differences for those who received either parenting or agribusiness training alone (parenting-only: IRR=0.78, 95% CI 0.36 to 1.68; agribusiness-only: IRR=1.29, 95% CI 0.62 to 2.70).

Parents in the parenting intervention villages also reported substantially reduced emotional abuse, though not those who received only the agribusiness training (combined: IRR=0.32, 95% CI 0.18 to 0.57; parenting-only: IRR=0.27, 95% CI 0.13 to 0.59; agribusiness-only: IRR=0.57, 95% CI 0.34 to 1.19). Children also reported reduced emotional abuse in the combined villages (IRR=0.27, 95% CI 0.08 to 0.89) with no child-reported differences in villages that received either intervention component alone (parenting-only: IRR=0.92, 95% CI 0.30 to 2.88; agribusiness-only: IRR=1.05, 95% CI 0.32 to 3.40).

There were parent-reported reductions of physical abuse in all three intervention groups (combined: IRR=0.32, 95% CI 0.17 to 0.62; parenting-only: IRR=0.34, 95% CI 0.18 to 0.66; agribusiness-only: IRR=0.52, 95% CI 0.28 to 1.00). However, children in the agribusiness-only villages reported substantially increased physical abuse (IRR=2.26, 95% CI 1.00 to 5.12). There were no differences for child-reported physical abuse in the parenting intervention villages (combined: IRR=0.50, 95% CI 0.19 to 1.30; parenting-only: IRR=0.99, 95% CI 0.34 to 2.35).

Children in the agribusiness-only villages also reported reduced positive parenting, (*D_w_*=−0.50, 95% CI −0.91 to 0.10), while there were borderline increases in positive parenting reported by parents receiving both intervention components (*D_w_*=0.26, 95% CI −0.10 to 0.62). There were no other differences for positive parenting reported by parents (parenting-only: *D_w_*=0.20, 95% CI −0.17 to 0.56; agribusiness-only: *D_w_*=−0.03, 95% CI −0.37 to 0.32) or children (combined: *D_w_*=−0.05, 95% CI −0.49 to 0.38; parenting-only: *D_w_*=−0.08, 95% CI −0.50 to 0.34).

Besides borderline effects for child-reported reduced child neglect reported in the combined villages (IRR=0.44, 95% CI 0.18 to 1.08), there were no other differences reported by parents (combined: IRR=1.21, 95% CI 0.51 to 2.84; parenting-only: IRR=0.68, 95% CI 0.28 to 1.65; agribusiness-only: IRR=0.72, 95% CI 0.31 to 1.71) or children (parenting-only: IRR=0.56, 95% CI 0.23 to 1.36; agribusiness-only: IRR=1.31, 95% CI 0.58 to 2.92).

There were no differences for sexual abuse reported by parents (combined: IRR=3.69, 95% CI 0.33 to 40.85; parenting-only: IRR=5.85, 95% CI 0.51 to 66.48; agribusiness-only: IRR=2.93, 95% CI 0.28 to 32.03) or children (combined: IRR=0.66, 95% CI 0.25 to 1.76; parenting-only: IRR=0.46, 95% CI 0.14 to 1.52; agribusiness-only: IRR=0.67, 95% CI 0.24 to 1.88).

### Secondary outcomes

Parents in the combined villages reported less endorsement of corporal punishment (*D_w_*=−0.43, 95% CI −0.79 to 0.07). There were also significant reductions in parent-reported child behaviour problems in all of the intervention arms (combined: *D_w_*=−0.41, 95% CI −0.77 to 0.05; parenting-only: *D_w_*=−0.47, 95% CI −0.84 to 0.11; agribusiness-only: *D_w_*=−0.43, 95% CI −0.77 to 0.08). There were also significant increases in household assets reported by parents who only received the agribusiness training (*D_w_*=0.57, 95% CI 0.08 to 1.06) and borderline increases in basic child necessities reported by children whose parents received the combined intervention (*D_w_*=0.43, 95% CI −0.07 to 0.79).

Borderline effect sizes were found in the combined villages for parent-report of reduced adult depression (*D_w_*=−0.43, 95% CI −0.91 to 0.06) and increased hours of child labour (*D_w_*=0.40, 95% CI −0.03 to 0.82). There were also borderline effects found in villages that received the agribusiness component for increased parent-report of maize production (combined: *D_w_*=0.54, 95% CI −0.04 to 1.12; agribusiness-only: *D_w_*=0.44, 95% CI −0.11 to 1.00) and measurements of mid-upper arm circumference for children aged 0–3 (combined: *D_w_*=0.45, 95% CI −0.10 to 0.99; agribusiness-only: *D_w_*=0.49, 95% CI −0.01 to 0.98).

There were no significant effects in the intervention arms on parenting stress, parent/child depression, positive child behaviour, child sexual behaviour, intimate partner violence, household hunger and child food consumption, child labour, agricultural production, family budgeting and any of the early child development outcomes. Analyses of parent and child alcohol use in the past month were not conducted due to extremely low reports of alcohol use at baseline (adults: n=4; children: n=3) and post-test (adults: n=13; children: n=0).

### Adverse effects

In addition to the adverse effects for increased physical abuse and reduced positive parenting reported by the subsample of children in the agribusiness-only villages, there were 32 cases of severe abuse reported at post-treatment assessment distributed equally across arms. These included reports of sexual assault (ie, either rape or attempted rape; n=22) or more than 30 instances of physical or emotional abuse (n=10) in the past month. In line with our ethical protocol, these cases were immediately referred to appropriate health and social services. One caregiver died prior to post-treatment assessments. This was unrelated to the study.

## Discussion

This study is the first cluster randomised trial to examine the combined and separate effect of parenting and economic strengthening programmes. It thus makes an important contribution to our knowledge and practice regarding reducing child maltreatment in LMICs. Results suggest that parenting programmes targeted at farmer groups may reduce the risk of violence against children in rural Tanzania. Parents in the intervention villages reported reductions in child maltreatment, with those in combined villages also reporting reduced endorsement of corporal punishment. It is also encouraging that children in villages receiving the combined intervention reported reduced overall maltreatment, thus corroborating adult reports. These results build on recent research in Burkina Faso that found reduced violence against children in families, where women received both an economic package and family coaching.^17^ Importantly, the current study also examined the effect of parenting without economic strengthening, allowing us to demonstrate that parenting interventions alone may be sufficient to reduce maltreatment, although with smaller effects. Results are particularly encouraging due to the high proportion of fathers and other male caregivers who are rarely included in studies,^12^ thus indicating that parenting programmes targeted at the community level through mixed-sex social structures may be effective at reaching fathers. The reductions in child maltreatment are also promising in the Tanzanian context, where authoritarian and harsh parenting are normative practices with high rates of violence against children reported in national surveys.^2^

With the exception of adult-reported borderline increases in positive parenting in the combined villages, the lack of improvements in positive parenting suggest that participating families may not be replacing harsh practices with alternative behaviours. This may have been due to the fact that the parenting programme was focused primarily on building knowledge and changing attitudes rather than the active practicing of skills, a core component for increasing positive parent involvement.^22^ Further research is required to examine whether or not the reduced harsh discipline is sustainable in the absence of positive replacement behaviours.

It is also encouraging that adults reported significant reductions in child behavioural difficulties in all of the intervention groups and borderline reductions in adult depression for combined and agribusiness-only groups. This suggests that both parenting and economic strengthening programmes may have a positive impact on child and adult outcomes in low-resource settings such as Tanzania. These results support longitudinal research indicating multiple pathways to improving child behaviour and parental mental health either indirectly via increased food security and household wealth, or directly by changing parenting behaviour.^23^

Findings indicating positive trends for increased economic well-being and food security in the combined and agribusiness-only villages suggest the utility of enhanced inputs and training in more efficient farming techniques. Although statistically not significant, villages that received agribusiness training showed marginal increases in measurements of infant and toddler growth, as well as marginal effects for child-reported reduced household hunger in the combined villages. These results may have been linked to marginal increases maize production as a cash crop which subsequently reduced food and financial insecurity. Interestingly, parent-report and child-report of household wealth was different in agribusiness-only and combined villages. Whereas parents reported increased number of general household assets in the agribusiness-only villages, children reported marginal increases in basic child necessities such as school supplies and clothing in the combined villages. This may be due to the parenting programme’s emphasis on parents’ responsibility towards child well-being and development. However, the marginal size of these effects requires additional research to examine the effect of agribusiness and parenting training on economic strengthening, food insecurity and financial prioritisation in families.

The harmful effects of increased physical abuse and reduced positive parenting reported by children (but not adults) in the agribusiness-only villages is particularly concerning. Possible mechanisms include reduced parent engagement and responsiveness due to increased agricultural activity and/or increased parent–child conflict due to demands on children to assist with farming activities or caregiving of younger children. These findings add to the growing body of research suggesting potential unintended consequences of economic strengthening programmes on child outcomes when delivered as a stand-alone intervention.^16^ For instance, an RCT of a microcredit loans programme in Bosnia found increased child labour and reduced school attendance for adolescents aged 16–19.^24^ Similarly, a cluster RCT of conditional cash transfers in Malawi found increased burden of household responsibilities and negative effects on mental health for adolescents.^25^ Furthermore, female caregivers who received only an economic intervention in Burkina Faso reported reduced quality of parent–child relationships at 24 month postassessment.^17^ Additionally, there is some evidence that economic strengthening programmes targeted at primarily male-led households may lead to increased risk of negative child-level outcomes in Uganda and Sri Lanka, as was the case in this study, although only when reported by children.^16^ Nonetheless, it is encouraging that these results were not evident in the villages that also received the parenting intervention, suggesting that the inclusion of parent training may mitigate potential harmful effects.

The null effects on early childhood development outcomes were not surprising given that the parenting intervention did not specifically address strategies for child development. This is one of the limitations of a universal parenting intervention for parents of children across the entire developmental spectrum. Additional content, specifically focused on the needs of infants and toddlers, may be necessary to increase effectiveness on improving the home environment in terms of cognitive stimulation, parental responsivity and child development.^26^

There were a number of limitations to this study. Assessments primarily relied on self-report data which are susceptible to social desirability reporting biases. It is possible that parents reported reduced child maltreatment outcomes because of their increased awareness that these were discouraged behaviours rather than because of a change in behaviour. Moreover, the divergent findings for reported physical abuse in the agribusiness-only intervention raise an important issue regarding the internal validity of results. Although the lack of convergence among most of the adult-reported and child-reported outcomes may be due to insufficient power, divergent patterns in child and adult reporting have been found elsewhere in RCTs on parenting in South Africa.^27^ Nonetheless, it is encouraging that there was corroboration between parent- and child-report on the primary outcome of reduced child maltreatment and emotional abuse by families receiving the combined parenting and agribusiness intervention.

Another limitation was low power and precision to detect intervention effects due to the small sample size of villages and recruited families in the cluster randomised design. This is an issue particularly for the child (n=176) and early childhood (n=138) samples which were lower than the total sample size (n=248), thus increasing the chance of a potential Type I error in the results. It was also not possible to directly compare adult-report and child-report data since adults reported on any child between the ages of 3 and 17 regardless of whether there was a child between the ages of 10 and 17 who also participated in the study. Future studies would benefit from a larger and more selective sample with parent–child pairs to allow more robust comparisons between adult-report and child-report data. Finally, results are limited by the fact that we were only able to conduct post-treatment assessments immediately after the interventions were delivered. Initially, the study was planned to have both an immediate postintervention and 4 month follow-up assessment. Due to delays in the inception of the study and in programme implementation, the initial immediate postintervention assessment was changed to a mid-treatment assessment resulting in only one postintervention assessment. A study with a longer-term follow-up would have also enabled the exploration of delayed treatment effects. For example, other studies have reported that parents may require more time to practice new approaches to discipline in order to implement them with consistency.^28^

There are a number of strengths of the study. It is rigorous compared with existing studies that investigate parenting interventions in LMICs.^10^ The cluster randomised design with both child-reported and parent-reported data allowed for a robust comparison of outcome effects with reasonable estimates of causality. The trial also provided a unique opportunity to investigate both the combined impact of parenting and economic strengthening interventions on reducing risks of violence against children, as well as the impact of these interventions alone. Furthermore, it was especially encouraging to find unusually high participation rates of male caregivers given widespread concern globally that fathers are often difficult to recruit and retain in parenting programmes.^29^ This resulted from the implementing agency’s strategic decision to nest the programme within farmer groups in rural communities, an existing social network that allowed for mixed-sex groups with a high proportion of fathers.

We recommend conducting a larger cluster RCT to provide more robust evidence on the effect of combining parenting and agribusiness training on reducing violence against children. Additional postassessments with a longer follow-up period would allow for mediation analyses to further understand the mechanisms driving changes in primary and secondary outcomes, especially concerning the interaction between parenting and economic outcomes. Observational assessments of parent–child interaction would also provide a more robust estimation of effects that are less susceptible to social desirability. Other potential study designs may include a factorial experimental design to provide further understanding of the differential effects of specific components of this complex family intervention. Furthermore, results suggest that a universal parenting programme delivered to families with children across the entire age range from 0 to 17 years may require additional early childhood development modules for those who have younger children. Lastly, studies examining the effects of economic strengthening interventions delivered without additional parenting components should be conducted with caution given the negative impacts reported by children in this study. At the very least, we recommend that researchers include assessments of parenting behaviour to examine possible harmful effects.

### Conclusion

This study makes a valuable contribution to our knowledge of the combined effect of parenting and economic strengthening programmes. Results indicate that while parenting and agribusiness training combined may be most effective at reducing risks of violence against children on a range of outcomes, parenting programmes delivered alone may also be effective. Moreover, findings also suggest that implementing an agricultural economic strengthening programme alone may, according to child reports, have some adverse effects on parent–child interaction leading to increased risk of violence against children. This is concerning given the pervasiveness of economic strengthening interventions in LMICs, and the inclusion of economic strengthening as part of recommended strategies to end violence against children.^14^ Nonetheless, these findings are particularly important given the barriers to engaging fathers experienced in other settings,^12^ and the limited evidence available regarding the effectiveness of complex community and family-based interventions in LMICs. Findings also provide an important foundation for future research examining the potential for complex interventions to accelerate positive impacts on multiple SDG targets across economic, social and environmental domains in Africa and beyond.^30^
