# Supplementary material for: Combining parenting and economic strengthening programmes to reduce violence against children: a cluster randomised controlled trial with predominantly male caregivers in rural Tanzania
Source: BMJ Glob Health. 2020 Jul 8;5(7):e002349. doi: 10.1136/bmjgh-2020-002349 (PMC7348478; doi:10.1136/bmjgh-2020-002349)
Supplement: Supplementary data [file bmjgh-2020-002349supp001.pdf]

Table 4. Summary of primary outcomes

Outcome[1]	Source[2]	No. Items	Range	Reliability[3]	Hypothesis[4]
Overall child maltreatment[5]	P	20	0 to 160	.79	All reduce
	C	24	0 to 192	.94	All reduce
Physical abuse	P	6	0 to 48	.72	All reduce
	C	8	0 to 64	.90	All reduce
Emotional abuse	P	9	0 to 72	.65	All reduce
	C	8	0 to 64	.85	All reduce
Sexual abuse	P	2	0 to 16	.79	All reduce
	C	2	0 to 16	.68	All reduce
Neglect	P	3	0 to 21	.54	All reduce
	C	6	0 to 32	.85	All reduce
Total positive parenting[6]	P	37	0 to 148	.79	All increase
	C	42	0 to 168	.76	All increase
Parent involvement	P	10	0 to 40	.81	All increase
	C	10	0 to 40	.77	All increase
Positive interaction	P	6	0 to 24	.79	All increase
	C	6	0 to 24	.79	All increase
Poor supervision	P	10	0 to 40	.65	All reduce
	C	10	0 to 40	.62	All reduce
Inconsistent discipline	P	6	0 to 24	.36	All reduce
	C	6	0 to 24	.47	All reduce
Effective discipline	P	5	0 to 20	.50	All increase
	C	6	0 to 24	.49	All increase
Harsh parenting	C	4	0 to 16	.67	All reduce

[1]All items summed unless otherwise specified; [2]P = Parent-report, C = child-report; [3]Cronbach alpha at baseline; [4]Intervention arms hypothesised direction of effect; [5]ISPCAN Child Abuse Screening Tool; [6]Alabama Parenting Questionnaire.

Table 5. Summary of secondary outcomes

Outcome[1]	Source[2]	No. Items	Range	Reliability[3]	Hypothesis[4]
**Psychosocial outcomes**					
Corporal punishment endorsement[5]	P	1	0/1	-	All reduce
	C	1	0/1	-	All reduce
Parenting stress[6]	P	18	0 to 72	.77	All reduce
Parent depression[7]	P	20	0 to 60	.89	All reduce
Child depression[8]	C	10	0 to 20	.64	All reduce
Intimate partner violence exposure[9]	P	10	0 to 80	.65	All reduce
Parent use of alcohol[10]	P	1	-	-	All reduce
Child use of alcohol[10]	C	1	-	-	All reduce
Child behaviour problems[11]	P	20	0 to 40	.70	All reduce
	C	20	0 to 40	.68	All reduce
Child prosocial behaviour[12]	P	10	0 to 20	.73	All increase
	C	10	0 to 20	.60	All increase
Child sexual behaviour[13]	C	2	-	-	All reduce
**Economic outcomes**					
Household hunger[14]	P	9	0 to 9	.88	Agri reduce
	C	9	0 to 9	.89	Agri reduce
Child food consumption[15]	C	1	0 to 3	-	Agri increase
Household assets[16]	P	12	0 to 12	.51	Agri increase
Basic child necessities[17]	C	9	0 to 9	.81	Agri increase
Child labour hours[18]	C	7	0 to 126	-	Agri increase
Agricultural maize production[19]	P	1	0 to 72	-	Agri increase
Attitudes toward family budgeting[20]	P	5	0 to 20	.28	All increase
**Early childhood outcomes**					
Stimulation and responsiveness[21]	P/O	40	0 to 40	.76	None[26]
Child development[22]	O	25	0 to 50	.80	None[26]
Child height in centimetres[23]	D	-	-	-	Agri increase
Child weight in kilograms[24]	D	-	-	-	Agri increase
Mid-upper arm circumference[25]	D	-	-	-	Agri increase

[1]All items summed unless otherwise specified; [2]P = Parent-report, C = child-report, P/O = Combined parent- and observation, O = Observation, D = Direct measurement; [3]Cronbach alpha at baseline; [4]Intervention arms hypothesised direction of effect; [5]Dichotomised item from the UNICEF Multiple Indicator Survey; [6]Parenting Stress Scale; [7]Centre for Epidemiological Studies Depression Scale; [8]Child Depression Inventory-Short Form; [9]Conflict Tactics Scale-Short Form; [10]Alcohol consumption in previous month; [11]Strengths and Difficulties Questionnaire; [12]Strengths and Difficulties Questionnaire Prosocial Behaviour subscale plus 5 items for *Heshima* (i.e., respectful behaviour); [13]South African National Survey of HIV and Risk Behaviour; [14]Hunger Questionnaire; [15]Number of meals in past week; [16]Items from UNICEF Multiple Indicator Survey; [17]Basic Child Necessities Scale; [18]Total numbers worked in past month; [19]Total number of 90 kilogram bags of maize produced in past month; [20]Household Income and Labour Dynamics Scale; [21]HOME Inventory; [22]Ages and Stages Questionnaire; [23]Measured using length board; [24]Measured using standard beam balance; [25]Mid-upper arm circumference; [26]No expected effect due to lack of content on early childhood development and adolescent sexual behaviour.

Table 6. Adult- and child-report of secondary outcomes using an intention-to-treat analysis and adjusting for differences at baseline.

	Parent-report (*N* = 248)					Child-report (*N* = 176)			
	Pre M (SD)	Post M (SD)	ES[1]	95% CI		Pre M (SD)	Post M (SD)	ES[1]	95% CI
Attitudes supporting corporal punishment[2]					Attitudes supporting corporal punishment[2]				
A+P	**2.92 (1.36)**	**1.53 (1.67)**	**-0.43**	**-0.79 to -0.07**	A+P	1.56 (1.56)	1.53 (1.44)	0.18	-0.18 to 0.54
A-only	2.17 (1.57)	1.84 (1.74)	-0.09	-0.43 to 0.26	A-only	1.80 (1.41)	1.73 (1.52)	0.30	-0.04 to 0.65
P-only	2.79 (1.35)	1.89 (1.65)	-0.18	-0.56 to 0.18	P-only	1.61 (1.35)	1.68 (1.49)	0.25	-0.11 to 0.62
Controls	2.53 (1.50)	2.09 (1.68)			Controls	1.92 (1.55)	1.32 (1.40)		
Adult depression[3]					Child depression[4]				
A+P	*13.80 (10.65)*	*10.09 (8.84)*	*-0.43*	*-0.91 to 0.06*	A+P	2.00 (2.05)	1.69 (1.69)	-0.09	-0.52 to 0.35
A-only	*13.92 (10.19)*	*10.54 (9.27)*	*-0.36*	*-0.82 to 0.10*	A-only	1.98 (2.33)	2.00 (2.26)	0.11	-0.30 to 0.51
P-only	14.95 (10.82)	11.88 (9.13)	-0.24	-0.72 to 0.24	P-only	1.40 (1.39)	1.38 (1.21)	-0.06	-0.49 to 0.36
Controls	15.12 (10.73)	14.00 (11.15)			Controls	2.49 (3.36)	1.90 (2.59)		
Parenting stress[5]					Child sexual behaviour[6]			ORR	
A+P	23.97 (8.90)	19.24 (10.92)	-0.18	-0.83 to 0.46	A+P	**28.63 (23.84)**	**20.48 (19.68)**	0.52	0.22 to 1.28
A-only	24.67 (8.66)	21.13 (10.08)	-0.05	-0.67 to 0.57	A-only	**25.16 (20.67)**	**23.25 (20.36)**	0.56	0.2 to 1.61
P-only	25.95 (10.24)	21.08 (9.56)	-0.08	-0.72 to 0.55	P-only	**26.45 (20.14)**	**24.48 (22.76)**	0.75	0.32 to 1.77
Controls	25.30 (9.33)	21.29 (10.74)			Controls	**27.31 (27.47)**	**23.76 (18.53)**	0.76	0.26 to 2.22
Child behaviour problems[7]					Child behaviour problems[7]				
A+P	**8.75 (5.34)**	**7.69 (4.18)**	**-0.41**	**-0.77 to -0.05**	A+P	7.09 (4.75)	7.56 (4.91)	-0.02	-0.55 to 0.52
A-only	**8.42 (5.46)**	**7.51 (3.84)**	**-0.43**	**-0.77 to -0.08**	A-only	6.26 (4.30)	7.39 (5.23)	-0.04	-0.45 to 0.53
P-only	**7.91 (4.95)**	**7.12 (4.48)**	**-0.47**	**-0.84 to -0.11**	P-only	6.68 (3.64)	8.74 (5.12)	0.24	-0.28 to 0.75
Controls	9.02 (5.05)	9.43 (5.29)			Controls	7.05 (5.69)	7.19 (4.62)		
Positive child behaviour[8]					Positive child behaviour[8]				
A+P	17.05 (3.42)	17.69 (3.63)	0.22	-0.29 to 0.72	A+P	18.62 (1.67)	17.93 (2.51)	0.08	-0.28 to 0.44
A-only	17.54 (2.83)	17.32 (3.90)	0.10	-0.39 to 0.59	A-only	18.04 (2.43)	18.18 (2.57)	0.20	-0.15 to 0.54
P-only	17.45 (2.86)	18.08 (2.82)	0.24	-0.26 to 0.75	P-only	18.63 (1.98)	17.76 (2.08)	0.06	-0.30 to 0.43
Controls	17.78 (2.83)	16.98 (4.17)			Controls	18.54 (2.38)	17.61 (3.43)		
Intimate partner violence[9]									
A+P	1.36 (3.18)	1.48 (7.07)	0.08	-0.28 to 0.44					
A-only	1.13 (4.77)	0.93 (3.66)	0.03	-0.31 to 0.38					
P-only	1.41 (5.57)	1.29 (5.65)	0.06	-0.30 to 0.43					
Controls	1.50 (4.92)	0.98 (2.28)							

[1]Hedges D_w effect sizes for positive parenting and attitudes supporting corporal punishment based on comparisons of post- assessment scores for each treatment group with the control group using linear-mixed effects models with restricted maximum likelihood (REML) and controlling for baseline differences; [2]Dichotomised item from the UNICEF Multiple Indicator Survey; [3]Centre for Epidemiological Studies Depression Scale; [4]Child Depression Inventory-Short Form; [5]Parenting Stress Scale; [6]South African National Survey of HIV and Risk

Behaviour; [7]Strengths and Difficulties Questionnaire; [8]Strengths and Difficulties Questionnaire Prosocial Behaviour subscale plus 5 items for *Heshima* (i.e., respectful behaviour); [9]Revised Conflict Tactics Scale-Short Form; A+P = Agribusiness plus Parenting; A-only = Agribusiness-only; P-only = Parenting-only; Significant effects in bold (95%CI not crossing 0); Borderline effects in italics (95%CI crossover within ±0.10 of 0).

Table 7. Adult- and child-report of economic outcomes using an intention-to-treat analysis and adjusting for differences at baseline.

Parent-report (*N* = 248)					Child-report (*N* = 176)				
	Pre M (SD)	Post M (SD)	ES	95% CI		Pre M (SD)	Post M (SD)	ES	95% CI
Household hunger					Household hunger				
A+P	3.43 (2.71)	2.28 (2.18)	-0.34	-0.99 to 0.31	A+P	*1.82 (2.65)*	*0.76 (1.42)*	*-0.39*	*-0.87 to 0.09*
A-only	3.82 (2.67)	2.71 (2.52)	-0.25	-0.88 to 0.37	A-only	1.74 (2.59)	1.09 (2.08)	-0.14	-0.58 to 0.30
P-only	3.84 (3.02)	3.25 (2.90)	0.00	-0.64 to 0.63	P-only	2.08 (2.52)	0.85 (1.44)	-0.29	-0.75 to 0.17
Controls	3.85 (3.07)	3.09 (2.69)			Controls	2.13 (2.63)	1.16 (2.02)		
Household wealth					Basic necessities scale				
A+P	3.25 (1.32)	3.64 (1.17)	0.33	-0.12 to 0.84	A+P	*5.98 (2.67)*	*6.42 (1.99)*	*0.43*	*-0.07 to 0.79*
A-only	**3.56 (1.51)**	**4.09 (1.63)**	**0.57**	**0.08 to 1.06**	A-only	6.73 (2.38)	6.30 (2.53)	0.16	-0.18 to 0.50
P-only	2.79 (1.50)	3.10 (1.72)	0.22	-0.28 to 0.73	P-only	7.26 (1.87)	6.59 (2.35)	0.09	-0.16 to 0.57
Controls	3.22 (1.39)	3.11 (1.59)			Controls	7.03 (2.53)	6.36 (3.00)		
Child labour – total hours in the past month					Child labour – total hours in the past month				
A+P	*2.08 (1.17)*	*2.53 (1.34)*	*0.40*	*-0.03 to 0.82*	A+P	2.08 (1.17)	2.53 (1.34)	-0.18	-0.57 to 0.22
A-only	1.74 (1.11)	1.98 (1.27)	0.05	-0.36 to 0.46	A-only	1.74 (1.11)	1.98 (1.27)	-0.01	-0.38 to 0.36
P-only	1.77 (0.99)	1.91 (1.44)	-0.02	-0.44 to 0.41	P-only	1.77 (0.99)	1.91 (1.44)	0.04	-0.35 to 0.43
Controls	1.75 (1.20)	1.92 (1.33)			Controls	1.75 (1.20)	1.92 (1.33)		
Agricultural assets					Child food consumption in past week				
A+P	24.55 (24.50)	25.19 (18.77)	0.08	-0.42 to 0.58	A+P	2.58 (0.56)	2.50 (0.71)	1.06	0.84 to 1.34
A-only	19.50 (15.69)	21.97 (16.10)	0.13	-0.36 to 0.61	A-only	2.62 (0.49)	2.45 (0.78)	1.05	0.84 to 1.31
P-only	16.80 (16.84)	20.17 (20.74)	0.07	-0.43 to 0.57	P-only	2.52 (0.54)	2.19 (0.87)	0.93	0.73 to 1.19
Controls	20.28 (20.15)	20.23 (20.42)			Controls	2.62 (0.52)	2.35 (0.78)		
Crop yield - maize									
A+P	*5.18 (5.33)*	*8.36 (6.65)*	*0.54*	*-0.04 to 1.12*					
A-only	*6.89 (7.14)*	*9.57 (7.81)*	*0.44*	*-0.11 to 1.00*					
P-only	6.05 (8.56)	8.02 (10.92)	0.36	-0.21 to 0.94					
Controls	5.18 (5.33)	8.36 (6.65)							
Family budgeting									
A+P	12.80 (2.46)	13.78 (2.99)	-0.03	-0.38 to 0.33					
A-only	13.74 (2.22)	14.51 (2.84)	0.16	-019 to 0.50					
P-only	12.66 (2.09)	13.88 (2.51)	0.05	-0.32 to 0.41					
Controls	13.07 (2.25)	13.88 (2.55)							

[1] Hedges D_w effect sizes based on comparisons of post-test scores for each treatment group with the control group using linear-mixed effects models with restricted maximum likelihood (REML) and controlling for baseline differences; A+P = Agribusiness plus Parenting; A-only = Agribusiness-only; P-only = Parenting-only; Significant effects in bold (95%CI not crossing 0); borderline effects in italics (95%CI crossover within ±0.10 of 0).

Table 8. Parent-report of early childhood outcomes using an intention-to-treat analysis and adjusting for differences at baseline (*N* = 134).

	Pre M (SD)	Post M (SD)	ES[1]	95% CI
Home environment stimulation and responsivity				
A+P	26.84 (4.66)	30.06 (5.02)	-0.01	-0.38 to 0.34
A-only	26.94 (5.24)	30.21 (4.25)	-0.04	-0.41 to 0.32
P-only	25.36 (4.45)	29.21 (6.16)	-0.12	-0.51 to 0.27
Controls	24.66 (5.67)	30.13 (4.35)		
Early childhood development				
A+P	26.42 (5.12)	28.50 (4.34)	0.09	-0.40 to 0.57
A-only	27.28 (7.44)	27.48 (7.16)	-0.2	-0.64 to 0.24
P-only	24.10 (7.66)	26.74 (5.98)	-0.25	-0.74 to 0.24
Controls	25.03 (7.65)	27.61 (6.34)		
Child upper-arm circumference				
A+P	*15.21 (1.28)*	*16.00 (1.18)*	*0.45*	*-0.10 to 0.99*
A-only	*15.53 (1.55)*	*16.10 (0.98)*	*0.49*	*-0.01 to 0.98*
P-only	14.66 (1.48)	15.03 (1.00)	-0.18	-0.73 to 0.38
Controls	14.67 (1.94)	15.17 (1.50)		
Child height				
A+P	10.26 (3.00)	12.65 (2.14)	0.12	-0.42 to 0.66
A-only	10.09 (2.45)	12.49 (2.15)	0.08	-0.41 to 0.56
P-only	9.84 (5.49)	11.65 (1.99)	-0.36	-0.91 to 0.19
Controls	9.11 (3.15)	12.00 (2.16)		
Child weight				
A+P	76.57 (11.07)	86.75 (7.33)	0.11	-0.39 to 0.60
A-only	74.94 (13.12)	85.28 (7.55)	-0.02	-0.47 to 0.43
P-only	73.40 (9.72)	83.05 (7.55)	-0.30	-0.81 to 0.20
Controls	71.41 (14.86)	83.61 (8.95)		

[1] Hedges D_w effect sizes based on comparisons of post-test scores for each treatment group with the control group using linear-mixed effects models with restricted maximum likelihood (REML) and controlling for baseline differences; A+P = Agribusiness plus Parenting; A-only = Agribusiness-only; P-only = Parenting-only; Significant effects in bold (95%CI not crossing 0); borderline effects in italics (95%CI crossover within ±0.10 of 0).

Table 9: CONSORT 2010 checklist for reporting a cluster randomised trial

Section/Topic	Item No	Standard Checklist item	Extension for cluster designs	Page No *
**Title and abstract**				
	1a	Identification as a randomised trial in the title	Identification as a cluster randomised trial in the title	3
	1b	Structured summary of trial design, methods, results, and conclusions (for specific guidance see CONSORT for abstracts)[1,2]	See table 2	3
**Introduction**				5-8
**Background and objectives**	2a	Scientific background and explanation of rationale	Rationale for using a cluster design	5, 7-8
	2b	Specific objectives or hypotheses	Whether objectives pertain to the the cluster level, the individual participant level or both	8
**Methods**				
**Trial design**	3a	Description of trial design (such as parallel, factorial) including allocation ratio	Definition of cluster and description of how the design features apply to the clusters	9
	3b	Important changes to methods after trial commencement (such as eligibility criteria), with reasons		13, 25
**Participants**	4a	Eligibility criteria for participants	Eligibility criteria for clusters	9-10
	4b	Settings and locations where the data were collected		9
**Interventions**	5	The interventions for each group with sufficient details to allow replication, including how and when they were actually administered	Whether interventions pertain to the cluster level, the individual participant level or both	10
**Outcomes**	6a	Completely defined pre-specified primary and secondary outcome measures, including how and when they were assessed	Whether outcome measures pertain to the cluster level, the individual participant level or both	12-13
	6b	Any changes to trial outcomes after the trial commenced, with reasons		N/A
**Sample size**	7a	How sample size was determined	Method of calculation, number of clusters(s) (and whether equal or unequal cluster sizes are assumed), cluster size, a coefficient of intracluster	11

			correlation (ICC or *k*), and an indication of its uncertainty	
	7b	When applicable, explanation of any interim analyses and stopping guidelines		N/A
**Randomisation:**				11
**Sequence generation**	8a	Method used to generate the random allocation sequence		
	8b	Type of randomisation; details of any restriction (such as blocking and block size)	Details of stratification or matching if used	N/A
**Allocation concealment mechanism**	9	Mechanism used to implement the random allocation sequence (such as sequentially numbered containers), describing any steps taken to conceal the sequence until interventions were assigned	Specification that allocation was based on clusters rather than individuals and whether allocation concealment (if any) was at the cluster level, the individual participant level or both	11
**Implementation**	10	Who generated the random allocation sequence, who enrolled participants, and who assigned participants to interventions	Replace by 10a, 10b and 10c	11
	10a		Who generated the random allocation sequence, who enrolled clusters, and who assigned clusters to interventions	11
	10b		Mechanism by which individual participants were included in clusters for the purposes of the trial (such as complete enumeration, random sampling)	11
	10c		From whom consent was sought (representatives of the cluster, or individual cluster members, or both), and whether consent was sought before or after randomisation	11
**Blinding**	11a	If done, who was blinded after assignment to interventions (for example, participants, care providers, those assessing outcomes) and how		11
	11b	If relevant, description of the similarity of interventions		10

**Statistical methods**	12a	Statistical methods used to compare groups for primary and secondary outcomes	How clustering was taken into account	13-14
	12b	Methods for additional analyses, such as subgroup analyses and adjusted analyses		14
**Results**				14-21
**Participant flow (a diagram is strongly recommended)**	13a	For each group, the numbers of participants who were randomly assigned, received intended treatment, and were analysed for the primary outcome	For each group, the numbers of clusters that were randomly assigned, received intended treatment, and were analysed for the primary outcome	14 (and supplemental file)
	13b	For each group, losses and exclusions after randomisation, together with reasons	For each group, losses and exclusions for both clusters and individual cluster members	14 (and supplemental file)
**Recruitment**	14a	Dates defining the periods of recruitment and follow-up		9-10
	14b	Why the trial ended or was stopped		N/A
**Baseline data**	15	A table showing baseline demographic and clinical characteristics for each group	Baseline characteristics for the individual and cluster levels as applicable for each group	15-16 (and supplemental file)
**Numbers analysed**	16	For each group, number of participants (denominator) included in each analysis and whether the analysis was by original assigned groups	For each group, number of clusters included in each analysis	14 (and supplemental file)
**Outcomes and estimation**	17a	For each primary and secondary outcome, results for each group, and the estimated effect size and its precision (such as 95% confidence interval)	Results at the individual or cluster level as applicable and a coefficient of intracluster correlation (ICC or k) for each primary outcome	17-20 (and supplemental file)
	17b	For binary outcomes, presentation of both absolute and relative effect sizes is recommended		N/A
**Ancillary analyses**	18	Results of any other analyses performed, including subgroup analyses and adjusted analyses, distinguishing pre-specified from exploratory		N/A
**Harms**	19	All important harms or unintended effects in each group (for specific		19

		guidance see CONSORT for harms[3])		
**Discussion**				22-27
**Limitations**	20	Trial limitations, addressing sources of potential bias, imprecision, and, if relevant, multiplicity of analyses		24-25
**Generalisability**	21	Generalisability (external validity, applicability) of the trial findings	Generalisability to clusters and/or individual participants (as relevant)	22-25
**Interpretation**	22	Interpretation consistent with results, balancing benefits and harms, and considering other relevant evidence		22-24
**Other information**				
**Registration**	23	Registration number and name of trial registry		9
**Protocol**	24	Where the full trial protocol can be accessed, if available		9
**Funding**	25	Sources of funding and other support (such as supply of drugs), role of funders		28

** Note: page numbers optional depending on journal requirements*

Table 10: Extension of CONSORT for abstracts to reports of cluster randomised trials[1,2]

Item	Standard Checklist item	Extension for cluster trials	Check (Y/N)
**Title**	Identification of study as randomised	Identification of study as cluster randomised	Y
**Trial design**	Description of the trial design (e.g. parallel, cluster, non-inferiority)		Y
**Methods**			
**Participants**	Eligibility criteria for participants and the settings where the data were collected	Eligibility criteria for clusters	Y
**Interventions**	Interventions intended for each group		Y
**Objective**	Specific objective or hypothesis	Whether objective or hypothesis pertains to the cluster level, the individual participant level or both	Y
**Outcome**	Clearly defined primary outcome for this report	Whether the primary outcome pertains to the cluster level, the individual participant level or both	Y
**Randomization**	How participants were allocated to interventions	How clusters were allocated to interventions	Y
**Blinding (masking)**	Whether or not participants, care givers, and those assessing the outcomes were blinded to group assignment		Y
**Results**			
**Numbers randomized**	Number of participants randomized to each group	Number of clusters randomized to each group	Y
**Recruitment**	Trial status[1]		N/A
**Numbers analysed**	Number of participants analysed in each group	Number of clusters analysed in each group	Y
**Outcome**	For the primary outcome, a result for each group and the estimated effect size and its precision	Results at the cluster or individual participant level as applicable for each primary outcome	Y
**Harms**	Important adverse events or side effects		Y
**Conclusions**	General interpretation of the results		Y
**Trial registration**	Registration number and name of trial register		Y
**Funding**	Source of funding		Y

[1] Relevant to Conference Abstracts

REFERENCES

[1] Hopewell S, Clarke M, Moher D, Wager E, Middleton P, Altman DG, et al. CONSORT for reporting randomised trials in journal and conference abstracts. *Lancet* 2008, 371:281-283
[2] Hopewell S, Clarke M, Moher D, Wager E, Middleton P, Altman DG at al (2008) CONSORT for reporting randomized controlled trials in journal and conference abstracts: explanation and elaboration. *PLoS Med* 5(1): e20
[3] Ioannidis JP, Evans SJ, Gotzsche PC, O'Neill RT, Altman DG, Schulz K, Moher D. Better reporting of harms in randomized trials: an extension of the CONSORT statement. *Ann Intern Med* 2004; 141(10):781-788.
